# Early enteral nutrition combined with supplemental parenteral nutrition vs. total parenteral nutrition after pancreaticoduodenectomy: a retrospective and propensity score-matched analysis of postoperative complications

**DOI:** 10.3389/fnut.2025.1606500

**Published:** 2025-11-27

**Authors:** Jialing Li, Defu Hu, Jianjie Sheng, Zhiang Wang, Hexing Hang, Yudong Qiu, Dayu Chen, Xu Fu

**Affiliations:** 1 Department of Pancreatic and Metabolic Surgery, Nanjing Drum Tower Hospital Clinical College of Nanjing University of Chinese Medicine, Nanjing, Jiangsu, China; 2 Department of Pancreatic and Metabolic Surgery, Nanjing Drum Tower Hospital, Affiliated Hospital of Medical School, Nanjing University, Nanjing, Jiangsu, China; 3 Department of Pathology, Nanjing Drum Tower Hospital, Affiliated Hospital of Medical School, Nanjing University, Nanjing, Jiangsu, China; 4 Department of Pharmacy, Nanjing Drum Tower Hospital, Affiliated Hospital of Medical School, Nanjing University, Nanjing, Jiangsu, China

**Keywords:** pancreaticoduodenectomy, nutritional support strategy, severe complications, early enteral nutrition, supplemental parenteral nutrition

## Abstract

**Background:**

Postoperative nutritional support strategy after pancreaticoduodenectomy (PD) remains controversial. This retrospective study aims to evaluate early enteral nutrition (EEN) combined with supplemental parenteral nutrition (SPN) vs. parenteral nutrition (PN) as postoperative nutritional support, focusing on early clinical outcomes and postoperative complications in patients who underwent PD.

**Methods:**

Clinical data from consecutive patients who underwent PD between January 2022 and July 2024 were collected and analyzed in this retrospective study. The primary outcome was the incidence of postoperative complications. The secondary outcomes included specific postoperative complications, such as delayed gastric emptying (DGE), bile leak (BL), chyle leak (CL), acute pancreatitis (AP), postpancreatectomy hemorrhage (PPH), and infectious complications, compared between the two groups. A propensity score-matched (PSM) analysis was performed to balance baseline confounders between the groups.

**Results:**

According to perioperative nutritional protocols, 248 patients were included and divided into the EEN + SPN group (*n* = 116) and the PN group (*n* = 132). After PSM, baseline characteristics were balanced between the EEN + SPN group (*n* = 59) and the PN group (*n* = 59). No statistically significant differences were observed in the incidence of complications between the two groups, either before or after PSM (all *p >* 0.05). Before PSM, the overall incidence of severe postoperative complications was 10.1%. The EEN + SPN group demonstrated a significantly lower incidence of severe complications compared to the PN group both before and after PSM (*p* < 0.05). Analysis of secondary outcomes (which included a comparative analysis of detailed complications) revealed no significant differences between the groups.

**Conclusion:**

In conclusion, this study demonstrates that for patients at nutritional risk following PD, EEN + SPN is a safe and feasible nutritional support strategy and is associated with a significant reduction in the incidence of severe complications.

## Introduction

Pancreaticoduodenectomy (PD) is the definitive procedure indicated for both benign and malignant disease localized in the pancreatic head, distal bile ducts, duodenum, and jugular abdomen, with high morbidity and mortality, and a postoperative complication rate of 40–60% (1–3). Preoperatively, patients undergoing PD often present with disease-related malnutrition (DRM). Postoperatively, they experience a state of negative nitrogen balance due to extensive organ resection and surgical trauma, which further exacerbates malnutrition (4). The preoperative nutritional risk is closely related to the postoperative recovery and long-term survival of patients undergoing pancreatic resection surgery (5–7). Therefore, providing adequate nutritional support is considered crucial for reducing the incidence of postoperative complications following PD.

Several nutritional support options are available after PD, including early enteral nutrition (EEN) and parenteral nutrition (PN), but no gold-standard consensus exists. Studies suggest that EEN is thought to be a more economical and reliable strategy for enhancing immune function, maintaining intestinal structure and function, and reducing the risk of postoperative infectious complications (8, 9). The European Society for Clinical Nutrition and Metabolism (ESPEN) and the Enhanced Recovery After Surgery (ERAS) Society guidelines recommend the implementation of postoperative EEN or oral nutritional supplements following gastrointestinal surgery (10, 11). A meta-analysis indicated that EEN should be prioritized for patients who can tolerate gastrointestinal feeding. If gastrointestinal feeding is not feasible, a combination of EEN with supplementary PN is also regarded as a safe and effective nutritional strategy (9). PN provides comprehensive energy and protein supplementation. A recent review indicated that PN may reduce the incidence of clinically relevant pancreatic fistula (12). However, the optimal strategy for postoperative nutritional support after PD remains unclear due to conflicting evidence and the lack of standardized guidelines (8, 12, 13). The current guidelines lack strong evidence to support, and there are significant differences in recommendations among various guidelines, mostly based on expert opinion (14, 15). Given the ongoing controversy surrounding the choice of early nutritional support following PD, this study aims to explore the risk factors for postoperative complications and assess the impact of EEN combined with SPN vs. PN on the incidence of postoperative complications.

## Methods

### Study design, patient screening, and ethics statement

The medical data of consecutive patients who underwent PD between January 2020 and July 2024 in the Department of Pancreatic Surgery, Nanjing Drum Tower Hospital, were collected in this retrospective cohort study. Patients were classified into EEN combined with the SPN group and the PN group. Demographics, preoperative and postoperative laboratory tests, and postoperative complications were collected. This study was approved by the revised Declaration of Helsinki and approved by our hospital’s Ethics Committee (2024–786).

### Inclusion and exclusion criteria

The inclusion criteria were: (1) age ≥18 years, (2) complete clinical data, (3) underwent conventional PD, (4) no evidence of locally unresectable or other active cancers at diagnosis, and (5) Nutritional Risk Screening 2002 (NRS-2002) score ≥3.

The exclusion criteria were: (1) inflammatory bowel disease, (2) combined other organ resections, (3) severe preoperative infections, and (4) severe renal dysfunction.

### Surgical procedures and perioperative management

All PDs were operated by two experienced surgical teams (The same surgeons participated in both surgical teams, and surgeons were cross-trained). All patients underwent either a conventional Whipple’s procedure with digestive reconstruction by Child’s approach, which included pancreatic-intestinal (Blumgart anastomosis), biliointestinal (hepatic ducts and jejunum with consecutive anterior and posterior wall stitches), and gastroenterostomy (Billroth II anastomosis). Standard perioperative management was implemented for all patients. In this study, all patients received a prophylactic intravenous injection of ceftriaxone, 30 min prior to surgery, continuing for 48 h postoperatively. Drain amylase, bacterial smear, and microbiological culture with antibiotic sensitivity were conducted on postoperative days (PODs) 1, 3, 5, and 7. A full abdominal enhanced computed tomography (CT) scan was performed on POD 7. The clinical team made individualized decisions about drainage tube removal based on symptoms, CT results, and biochemical indicators. Patients identified as with nutritional risk preoperatively received oral intact protein-based enteral nutrition supplements, providing approximately 300–500 kcal/day for 3–5 days.

### Postoperative nutrition

In patients in the EEN + SPN group, a gastric tube was routinely placed intraoperatively. During surgery, nasojejunal nutrition tubes were placed in the jejunum to facilitate postoperative EN delivery. EN was initiated within 24 h following abdominal surgery according to standard protocols based on ESPEN guidelines. Within this time frame, a 5% glucose and 0.9% sodium chloride solution was administered at a rate of 1.25 to 1.67 mL/kg/h via the nasojejunal feeding tube. On POD 2, EN was provided at a low rate, 500 mL of enteral formula (providing 1 kcal/mL of energy, comprising 16% protein, 35% fat, and 49% carbohydrates), and 250 mL of 5% glucose and 0.9% sodium chloride solution were administered. On POD 3, 500–1,000 mL of EN was administered, and the PN formula was adjusted based on the amount of EN given to meet the remaining energy requirements.

In the PN group, the gastric tube was not routinely placed, and central venous catheters (CVCs) were placed after anesthesia. The PN included intravenous lipid emulsions, micronutrients, amino acids, glucose, and vitamins that were administered continuously, 24 h a day for up to 7 days from the first day after PD. The total caloric intake was fixed at 25–30 kcal/kg/d, the proportion of protein calories with a nitrogen ratio of 120–150:1. Clinical pharmacists adjusted the caloric and protein supplements, as well as fluid and electrolyte levels, based on the patient’s weight, laboratory indicators, and dietary intake. The PN was administered via CVCs. As the patient gradually resumed adequate oral intake, the PN was tapered off.

### Clinical data collection and definition of outcomes

The clinical data from medical records included demographics (age, sex, comorbidity, and body mass index); preoperative jaundice; preoperative biliary drainage; the Global Leadership Initiative on Malnutrition (GLIM) criteria; and preoperative laboratory data (total protein [TP], serum albumin [Alb], prealbumin [PA], hemoglobin [Hb], and bilirubin). Additionally, intra-operative variables were recorded, including operating time, volume of blood loss, vessel resection, pancreatic texture, and surgical method. Postoperative nutritional indices, such as TP, Alb, and Hb, were also assessed, along with the duration of postoperative hospital stay, hospitalization costs, and pathological diagnosis.

The primary outcome was the incidence of postoperative complications. If patients experienced multiple complications, they were recorded only once according to the highest-grade complication based on the Clavien-Dindo classification system, and severe complications were defined as grade≥III (16). The secondary outcomes encompassed a comparison of postoperative complications between the two groups, including DGE, BL, CL, AP, PPH, and infectious complications. Clinically relevant postoperative pancreatic fistula (CR-POPF, grade B/C), biliary leak (BL), postoperative acute pancreatitis (PPAP), delayed gastric emptying (DGE), chylous fistula (CL), and post-pancreatectomy hemorrhage (PPH) were diagnosed based on the International Study Group for Pancreatic Surgery (ISGPS) (17–21). Exploratory outcomes included postoperative nutritional indices TP, Alb, and Hb. The analysis also encompassed wound infection, bacteremia, intra-abdominal infection, urinary tract infection, and pneumonia (22).

### Statistical analysis

All statistical analyses were carried out using SPSS 27.0 software and R version 4.0. Categorical variables were reported as frequencies and percentages, and with the X^2^ or Fisher’s exact test applied for the comparison. Quantitative variables were characterized by mean±standard deviation or median (interquartile range, IQR) contingent upon the assessment of normal distribution, and *t*-test or Mann–Whitney U-test was used for the analyses. To assess the effect of nutrition therapy on clinical outcomes, logistic regression was utilized. Odds ratio (OR) with 95% confidence interval (CI) and *p*-values were documented. A *p*-value of less than 0.05 was considered statistically significant.

**Figure 1:**
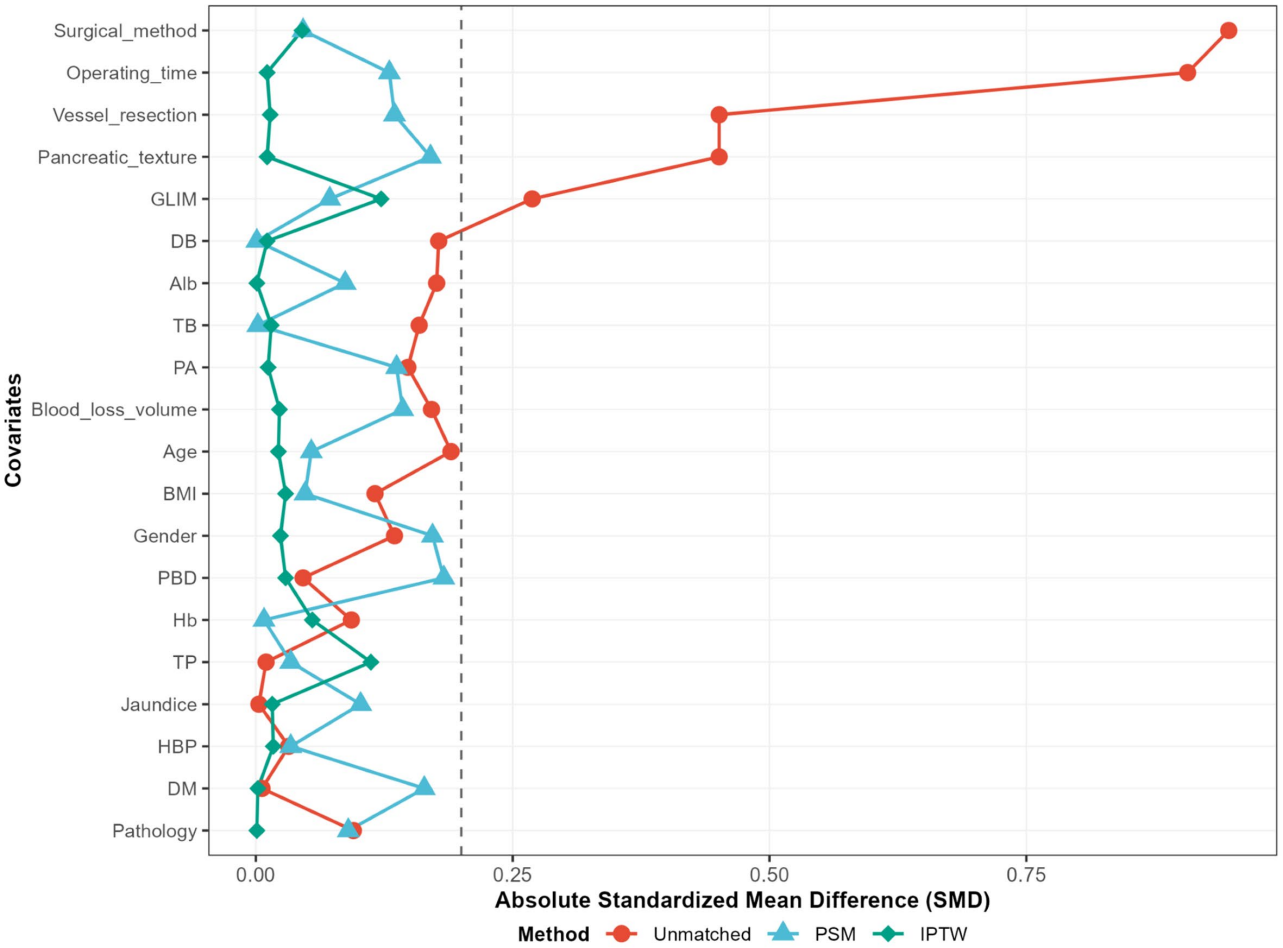
Standardized mean differences (SMD) of patients, patients after propensity score matching; PSM, propensity score matching; IPTW, inverse probability of treatement weighting; unmatched, raw data.

To mitigate potential confounding effects arising from baseline differences between the two cohorts, we implemented a propensity score-matched (PSM) analysis approach to adjust for baseline characteristics. We matched the two groups in a 1:1 ratio with a caliper width of 0.2. The standardized mean difference (SMD) of less than 0.2 was used to examine the degree of PSM. With an SMD threshold of <0.20, indicating a successful balance between groups and absence of significant inter-group differences (Figure 1). To assess the robustness of our findings, we conducted a sensitivity analysis using inverse probability of treatment weighting (model 2) to mitigate the impact of confounding variables and reduce the likelihood of selection bias. Among patients in the EEN + SPN group, we examined the association between POD 3 EN kcal/kg/d and postoperative complications using

logistic regression with restricted cubic splines (RCS). Splines were used for four knots at the 5th, 35th, 65th, and 95th percentiles of POD3EN to allow for potential non-linearity. A two-proportion Fisher’s exact test was performed using PASS 2025 software to evaluate statistical power (*α* = 0.05). The analysis included 116 patients in the treatment group (event proportion: 0.034) and 132 in the control group (event proportion: 0.160), yielding a proportion difference of −0.126. The resulting statistical power was 0.8818, indicating an 88.2% probability of detecting a true difference between groups and confirming the high sensitivity of the study design.

## Results

### Patient characteristics

A total of 248 patients who underwent PD were initially enrolled in the study. Patients were divided into two groups based on perioperative nutritional therapy. The characteristics of all patients are shown in Table 1. There were 116 (46.8%) patients in EEN + SPN group, 132 (53.2%) in PN group. The study cohort consisted of 143 males (57.7%) and 105 females (42.3%), with an average age of 66.5 ± 9.8 years. Before surgery, the levels of total direct bilirubin (DB) and bilirubin (TB) were 10.7 (2.3, 86.1) umol/L and 24.0 (10.5, 116.5) umol/L, respectively. A total of 111 (44.8%) were diagnosed with preoperative jaundice, and 69 (27.8%) underwent preoperative biliary drainage (PBD). Postoperative complications occurred in 173 patients (69.8%). Postoperative severe complications (Clavien-Dindo grade≥III) occurred in 25 patients (10.1%), intra-abdominal infections were observed in 103 patients (41.5%), and CR-POPF developed in 50 patients (20.2%).

**Table 1:** Clinical characteristics of all patients.

Characteristics	Total (*n* = 248)
Age (mean ± SD), years	66.5 ± 9.8
Sex, *n* (%)	
Male	143 (57.7)
Female	105 (42.3)
BMI (mean ± SD), kg/m^2^	22.6 ± 2.9
DM, *n* (%)	51 (20.6)
HBP, *n* (%)	124 (50.0)
Jaundice, *n* (%)	111 (44.8)
PBD, *n* (%)	69 (27.8)
GLIM, *n* (%)	156 (62.9)
TP (mean ± SD), g/L	64.3 ± 5.9
Alb (mean ± SD), g/L	38.0 ± 3.6
PA (mean ± SD), mg/L	193.6 ± 59.2
Hb (mean ± SD), g/L	121.2 ± 20.5
TB (median, IQR), μmol/L	24.0 (10.5, 116.5)
DB (median, IQR), μmol/L	10.7(2.3, 86.1)
Nutritional support, *n* (%)	
EEN + SPN	116 (46.8)
PN	132(53.2)
Operating time (median, IQR), min	300.0(240.0, 383.7)
Blood loss volume (median, IQR), mL	400.0 (200.0, 500.0)
Surgical method, *n* (%)	
PD	176 (71.0)
PPPD	72 (29.0)
Vessel resection, *n* (%)	
Yes	26 (10.5)
Pancreatic texture, *n* (%)	
Soft	140 (56.5)
Firm	108 (43.5)
Pathology diagnosis, *n* (%)	
PDAC	207 (83.5)
No-PDAC	41 (16.5)
Postoperative hospital stays (median, IQR), day	19.0 (14.0, 28.0)
Cost (median, IQR), dollars	114029.9 (96369.8, 138895.8)
Postoperative complications, *n* (%)	
Yes	173 (69.8)
No	75 (30.2)
Postoperative complications, *n* (%)	
CR-POPF	50 (20.2)
DGE	39 (15.7)
BL	24 (9.7)
CL	35 (14.1)
AP	5 (2.0)

Characteristics	Total (*n* = 248)
PPH	14 (5.6)
Abdominal infection	103 (41.5)
Pneumonia	5 (2.0)
Surgical site	5 (2.0)
Urinary tract infection	1 (0.4)
Bacteremia	11 (4.4)
Clavien-Dindo I, *n* (%)	22 (8.9)
Clavien-Dindo II, *n* (%)	126 (50.8)
Clavien-Dindo III–V, *n* (%)	25 (10.1)

BMI, body mass index; DM, diabetes mellitus; HBP, high blood pressure; PBD, preoperative biliary drainage; GLIM, the global leadership initiative on malnutrition criteria; TP, total protein; Alb, albumin; PA, prealbumin; Hb, hemoglobin; TB, total bilirubin; DB, direct bilirubin; EEN, early enteral nutrition; SPN, supplemental parenteral nutrition; PN, parenteral nutrition; PD, pancreaticoduodenectomy; PPPD, pylorus-preserving pancreaticoduodenectomy; PDAC, pancreatic duct adenocarcinoma; CR-POPF, clinically relevant postoperative pancreatic fistula (grade B/C); DGE, delayed gastric emptying; BL, biliary leak; CL, chylous fistula; AP, acute pancreatitis; PPH, post-pancreatectomy hemorrhage.

As shown in Table 2, the PN group had a longer operative time, a higher preoperative incidence of GLIM-defined malnutrition, and a substantial number of PPPD compared to the EEN + SPN group. Additionally, there were statistically significant differences in rates of vascular resection and pancreatic texture between the groups. To adjust for baseline confounders, a 1:1 PSM analysis was performed. After PSM, the standardized mean differences for all preoperative covariates were less than 0.20. The final cohort comprised the EEN + SPN group (59 patients) and the PN group (59 patients). All baseline characteristics were comparable after PSM. Details on sensitivity analyses are provided in the Supplementary Appendix, Supplemental Digital Content 1, https://10.6084/m9.figshare.28559741.

**Table 2:** Baseline characteristics in the unmatched and matching groups according to perioperative nutritional support modality.

Variables	Before PS matching				Variables	After PS matching			
	EEN + SPN (*n* = 116)	PN (*n* = 132)	*P*	SMD		EEN + SPN (*n* = 59)	PN (*n* = 59)	*P*	SMD
Age (mean ± SD), years	65.5 ± 9.5	67.3 ± 9.9	0.136	0.190	Age (mean ± SD), years	66.6 ± 9.0	66.1 ± 10.4	0.769	0.054
Gender, *n* (%) Male Female	71 45	72 60	0.289	0.135	Gender, *n* (%) Male Female	36 23	31 28	0.457	0.172
BMI (mean ± SD), kg/m^2^	22.8 ± 3.0	22.4 ± 2.8	0.365	0.116	BMI (mean ± SD), kg/m^2^	22.3 ± 2.9	22.2 ± 2.8	0.794	0.048
GLIM, *n* (%)	65 (56.0)	91 (68.9)	0.036	0.269	GLIM, *n* (%)	38 (64.4)	40 (67.8)	0.846	0.072
DM, *n* (%)	24 (20.7)	27 (20.5)	0.964	0.006	DM, *n* (%)	15 (25.4)	11 (18.6)	0.505	0.164
HBP, *n* (%)	57 (49.1)	67 (50.8)	0.799	0.032	HBP, *n* (%)	31 (52.5)	32 (54.2)	1.000	0.034
Jaundice, *n* (%)	52 (44.8)	59 (44.7)	0.984	0.003	Jaundice, *n* (%)	28 (47.5)	31 (52.5)	0.713	0.102
PBD, *n* (%)	31 (26.7)	38 (28.8)	0.717	0.046	PBD, *n* (%)	16 (27.1)	21 (35.6)	0.427	0.183
TP (mean ± SD), g/L	64.3 ± 6.4	64.3 ± 5.6	0.939	0.010	TP (mean ± SD), g/L	64.2 ± 7.2	64.0 ± 5.9	0.854	0.034
Alb (mean ± SD), g/L	38.3 ± 3.6	37.7 ± 3.5	0.133	0.176	Alb (mean ± SD), g/L	37.8 ± 3.9	37.4 ± 3.5	0.639	0.087
PA (mean ± SD), mg/L	199.9 ± 59.6	188.1 ± 58.6	0.134	0.148	PA (mean ± SD), mg/L	192.2 ± 58.8	184.6 ± 52.8	0.459	0.137
Hb (mean ± SD), g/L	122.2 ± 17.6	120.3 ± 22.7	0.460	0.093	Hb (mean ± SD), g/L	120.2 ± 18.1	120.7 ± 26.3	0.964	0.008
TB (median, IQR), μmol/L	21.5 (10.7, 137.8)	24.2 (10.2, 110.1)	0.574	0.159	TB (mean ± SD), μmol/L	84.2 ± 102.5	84.4 ± 98.9	0.991	0.002
DB (median, IQR), μmol/L	10.5 (2.2, 95.1)	12.0 (2.4, 77.8)	0.754	0.178	DB (mean ± SD), μmol/L	54.9 ± 71.5	54.9 ± 67.8	0.999	<0.001
Pancreatic texture, *n* (%) Soft Firm	52 (44.8) 64 (55.2)	88 (66.7) 44 (33.3)	<0.001	0.451	Pancreatic texture, *n* (%) Soft Firm	26 (44.1) 33 (55.9)	31 (52.5) 28 (47.5)	0.461	0.170
Pathology, *n* (%) PDAC Non-PDAC	99 (85.3) 17 (14.7)	108 (81.8) 24 (18.2)	0.456	0.095	Pathology, *n* (%) PDAC Non-PDAC	50 (84.7) 9 (15.3)	48 (81.3) 11 (18.7)	0.806	0.090
Vessel resection, *n* (%) Yes	4 (3.4)	22 (16.7)	<0.001	0.451	Vessel resection, *n* (%) Yes	3 (5.1)	5 (8.5)	0.714	0.135
Surgical method, *n* (%) PD PPPD	106 (91.4) 10 (8.6)	70 (53.0) 62 (47.0)	<0.001	0.947	Surgical method, *n* (%) PD PPPD	49 (83.0) 10 (17.0)	50 (85.0) 9 (15.0)	1.000	0.046
Operating time (mean ± SD), min	271.7 ± 73.6	351.9 ± 101.3	<0.001	0.907	Operating time (mean ± SD), min	291.2 ± 82.3	303.3 ± 106.7	0.483	0.130
Blood loss volume (median, IQR), mL	300 (200.0, 500.0)	400.0 (200.0, 600.0)	0.114	0.171	Blood loss volume (mean ± SD), mL	427.1 ± 300.5	472.2 ± 316.9	0.440	0.046

BMI, body mass index; DM, diabetes mellitus; HBP, high blood pressure; PBD, preoperative biliary drainage; TP, Total protein; Alb, albumin; PA, realbumin; Hb, hemoglobin; TB, total bilirubin; DB, direct bilirubin; EEN, early enteral nutrition; SPN , supplemental parenteral nutrition; TPN, total parenteral nutrition; PD, pancreaticoduodenectomy; PPPD, pylorus-preserving pancreaticoduodenectomy; PDAC, pancreatic duct adenocarcinoma.

### Outcomes

No statistically significant differences were observed in the incidence of complications between the two groups, either before or after PSM (all *p* > 0.05, Table 3). Before PSM, the incidence of severe postoperative complications was 3.4% in the EEN + SPN group, compared to 15.9% in the PN group (*p =* 0.002, Table 3). After PSM, the incidence of severe complications was 5.1% in the EEN + SPN group and 20.3% in the PN group (*p =* 0.027, Table 3). Both before and after PSM, the incidence of severe complications was significantly higher in the PN group. Before PSM, the incidence of intra-abdominal infection was significantly higher in the PN group (34.5% *vs.* 47.7%, *p* = 0.035). However, after PSM, the difference was no longer statistically significant (33.9% *vs.* 42.4%, *p* = 0.448). Both before and after PSM, there was no significant difference in CD-I complications between the EEN + SPN and PN groups (before PSM: 7.8% *vs.* 9.8%, *p* = 0.724; after PSM: 10.2% *vs.* 3.4%, *p* = 0.600). Similar results were observed for CD-II complications (before PSM: 54.3 *vs.* 47.7%, *p* = 0.364; after PSM: 55.9% *vs.*39.0%, *p* = 0.097) and CR-POPF (before PSM: 17.2% *vs.* 22.7%, *p* = 0.283; after PSM: 22.0% *vs.* 20.3%, *p* = 1.000). No significant differences were found in the remaining complications between the two groups (Table 3).

**Table 3:** Comparison of postoperative complications in the unmatched and matched groups according to perioperative nutritional support modality.

Variables	Before PS matching			After PS matching		
	EEN + SPN = 116	PN = 132	*P*	EEN + SPN = 59	PN = 59	*P*
Postoperative complications	76 (65.5%)	97 (73.5%)	0.221	42 (71.2%)	37 (62.7%)	0.434
CD-I	9 (7.7%)	13 (9.8%)	0.724	6 (10.2%)	2 (3.4%)	0.600
CD-II	63 (54.3%)	63 (47.7%)	0.364	33 (55.9%)	23 (39.0%)	0.097
CD-III–V	4 (3.4%)	21 (15.9%)	0.002	3 (5.1%)	12 (20.3%)	0.027
CR-POPF	20 (17.2%)	30 (22.7%)	0.283	13 (22.0%)	12 (20.3%)	1.000
DGE	20 (17.2%)	19 (14.4%)	0.539	12 (20.3%)	10 (16.9%)	0.813
BL	7 (6.0%)	17 (12.9%)	0.069	4 (6.8%)	7 (11.9%)	0.527
CL	14 (12.1%)	21 (15.9%)	0.386	10 (16.9%)	6 (10.2%)	0.420
AP	1 (0.9%)	4 (3.0%)	0.448	1 (1.7%)	2 (3.4%)	1.000
PPH	4 (3.4%)	10 (7.6%)	0.160	2 (3.4%)	4 (6.8%)	0.675
Abdominal infection	40 (34.5%)	63 (47.7%)	0.035	20 (33.9%)	25 (42.4%)	0.448
Pneumonia	2 (1.7%)	3 (2.3%)	1.000	2 (3.4%)	2 (3.4%)	1.000
Surgical site	1 (0.9%)	4 (3.0%)	0.448	0 (0%)	2 (3.4%)	0.476
Urinary tract infection	1 (0.9%)	0 (0%)	0.948	1 (1.7%)	0 (0%)	1.000
Bacteremia	3 (2.6%)	8 (6.1%)	0.185	2 (3.4%)	7 (11.9%)	0.165

CR-POPF, Clinically relevant postoperative pancreatic fistula (Grade B/C); DGE, delayed gastric emptying; BL, biliary leak; CL, chylous fistula; AP, acute pancreatitis; PPH, post-pancreatectomy hemorrhage.

**Figure 2:**
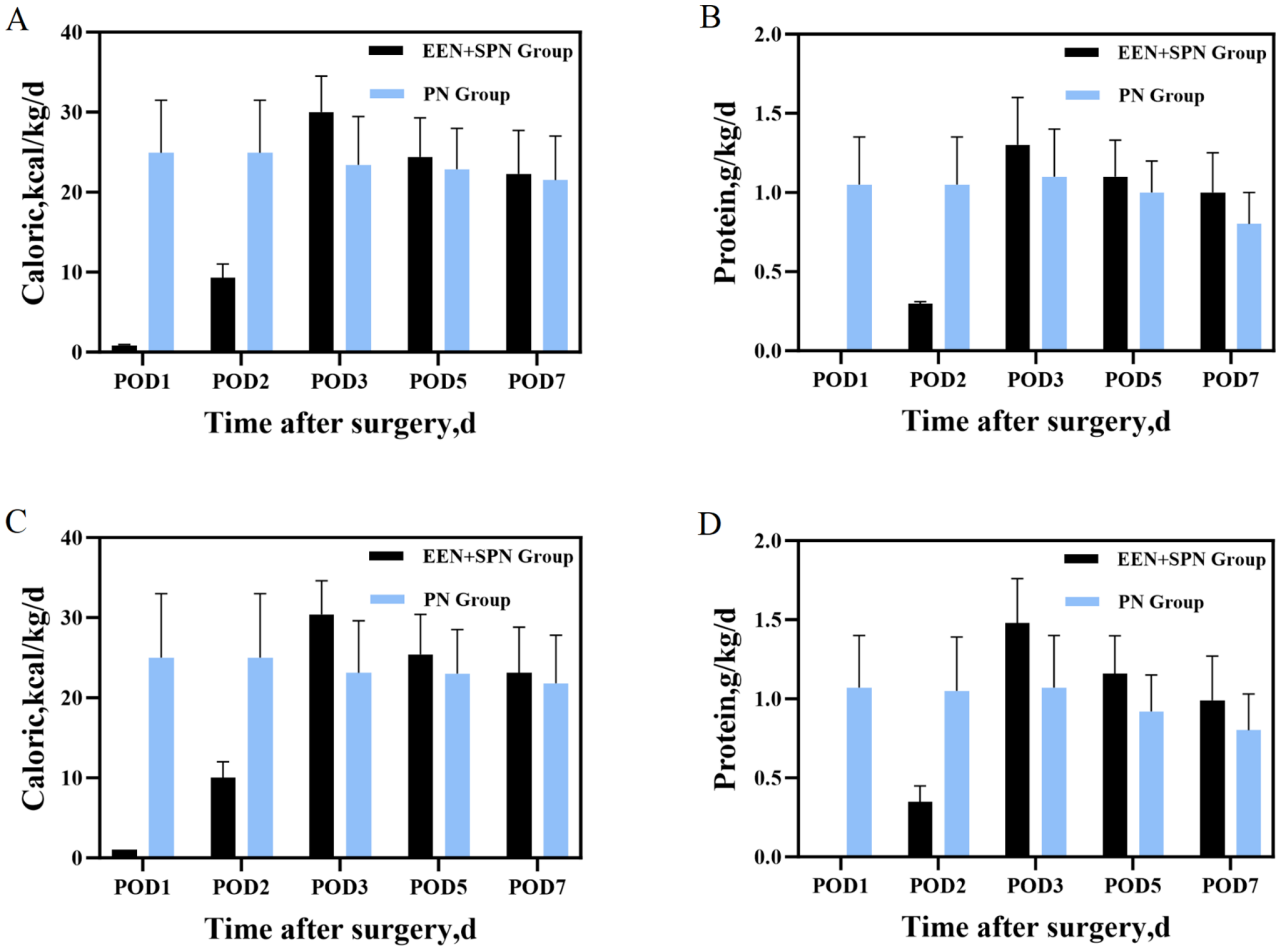
**(A)** Before PSM, mean daily caloric intake during the 7 days after surgery; **(B)** before PSM, mean daily protein intake during the 7 days after surgery; **(C)** after PSM mean daily caloric intake during the 7 days after surgery; **(D)** after PSM mean daily protein intake during the 7 days after surgery.

**Figure 3:**
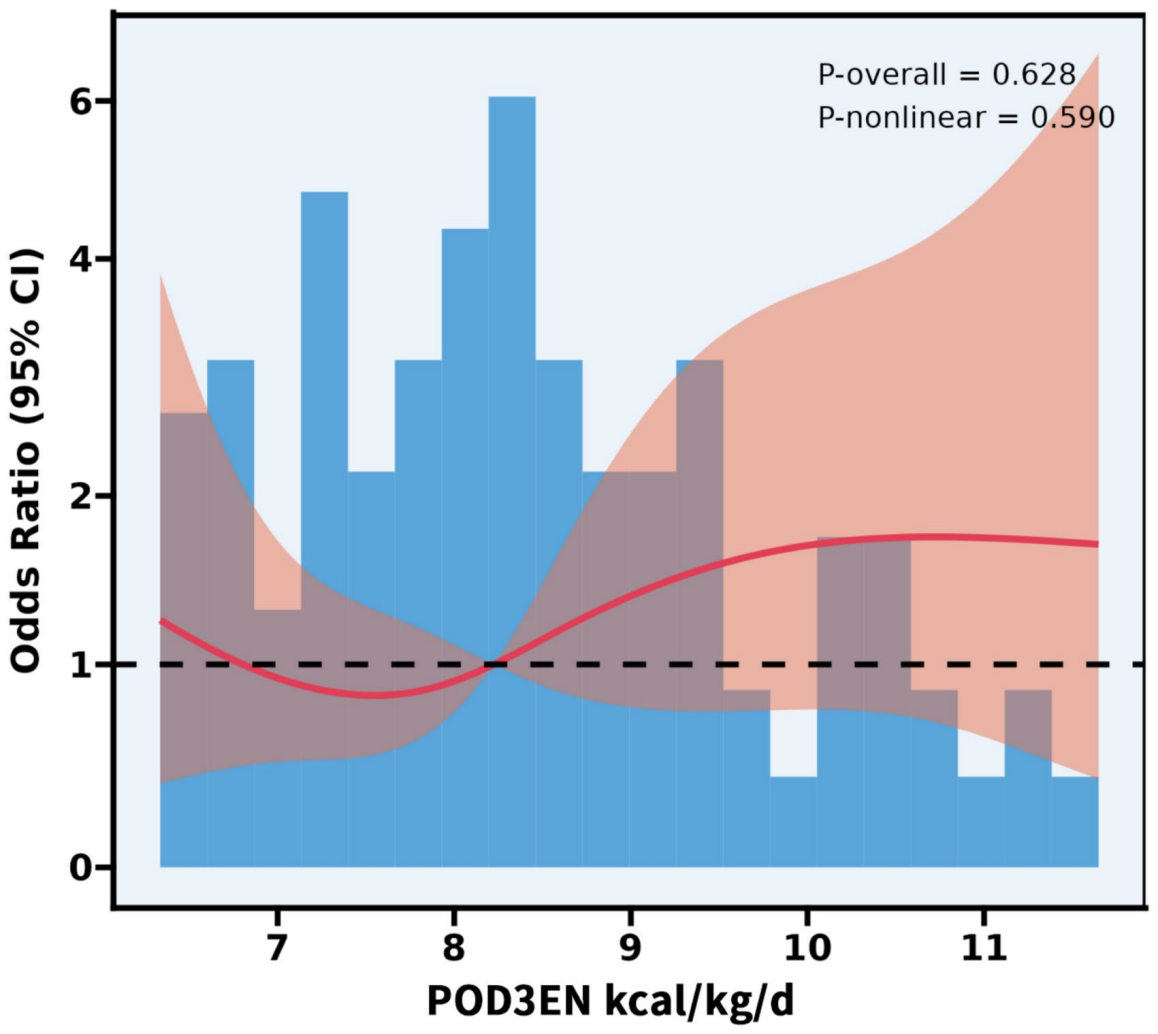
Association between POD3 EN kcal/kg/d and complications with the RCS function. Model with 4 knots located at 5th, 35th, 65th, and 95th percentiles. Y-axis presents the OR to present complications for any value of POD3 EN kcal/kg/d compared to individuals with reference vlaue (50th percentile) of POD3EN kcal/kg/d.

The body weight of the patients in this study ranged from 40 to 86.5 kg. On POD 3, patients in the EEN + SPN group received a mean (SD) energy intake of 30 (4.5) kcal/kg/d, in contrast to the PN group, which had a mean (SD) energy intake of 23.4 (6.0) kcal/kg/d. On POD 7, patients in the EEN + SPN group received a mean (SD) energy intake of 22.3 (5.5) kcal/kg/d, while those in the PN group received a mean (SD) energy intake of 21.5 (5.5) kcal/kg/d. Throughout this period, the mean (SD) protein intake was 1.3 (0.3), 1.0 (0.2) g/kg/day in the EEN + SPN group and 1.0 (0.2), 0.8 (0.2) g/kg/day in the PN group. Before and after PSM, the average daily energy and protein intake for both groups 1 week postoperatively is provided in Figure 2. The period on POD 3 represented a dynamic titration phase, with caloric intake for all patients stabilizing within the target range (25–30 kcal/kg/day) at POD 5. In a longitudinal analysis of patients receiving nutritional support, RCS regression found no evidence of a linear or non-linear dose–response relationship between enteral nutrition and the risk of complications. The results showed that neither the linear term (coefficient *=* −0.485, SE *=* 0.608, *p =* 0.427) nor the two non-linear spline terms (3.095, SE = 3.197, *p* = 0.335; −9.851, SE = 10.761, *p* = 0.362) were statistically significant, indicating no reliable evidence for either a linear or non-linear association. Further analysis using piecewise logistic regression (breakpoint = 8.224) to compare single-slope and dual-slope models showed no significant effects on either side of the breakpoint (<8.224: OR *=* 0.94, 95% CI: 0.60–1.47, *p =* 0.784; ≥8.224: OR = 1.18, 95% CI: 0.79–1.76, *p* = 0.415). Additionally, dose-based ROC analysis demonstrated limited discriminative ability (AUC *=* 0.550, 95% CI: 0.439–0.661). (Figure 3, Supplementary Table S3).

**Figure 4:**
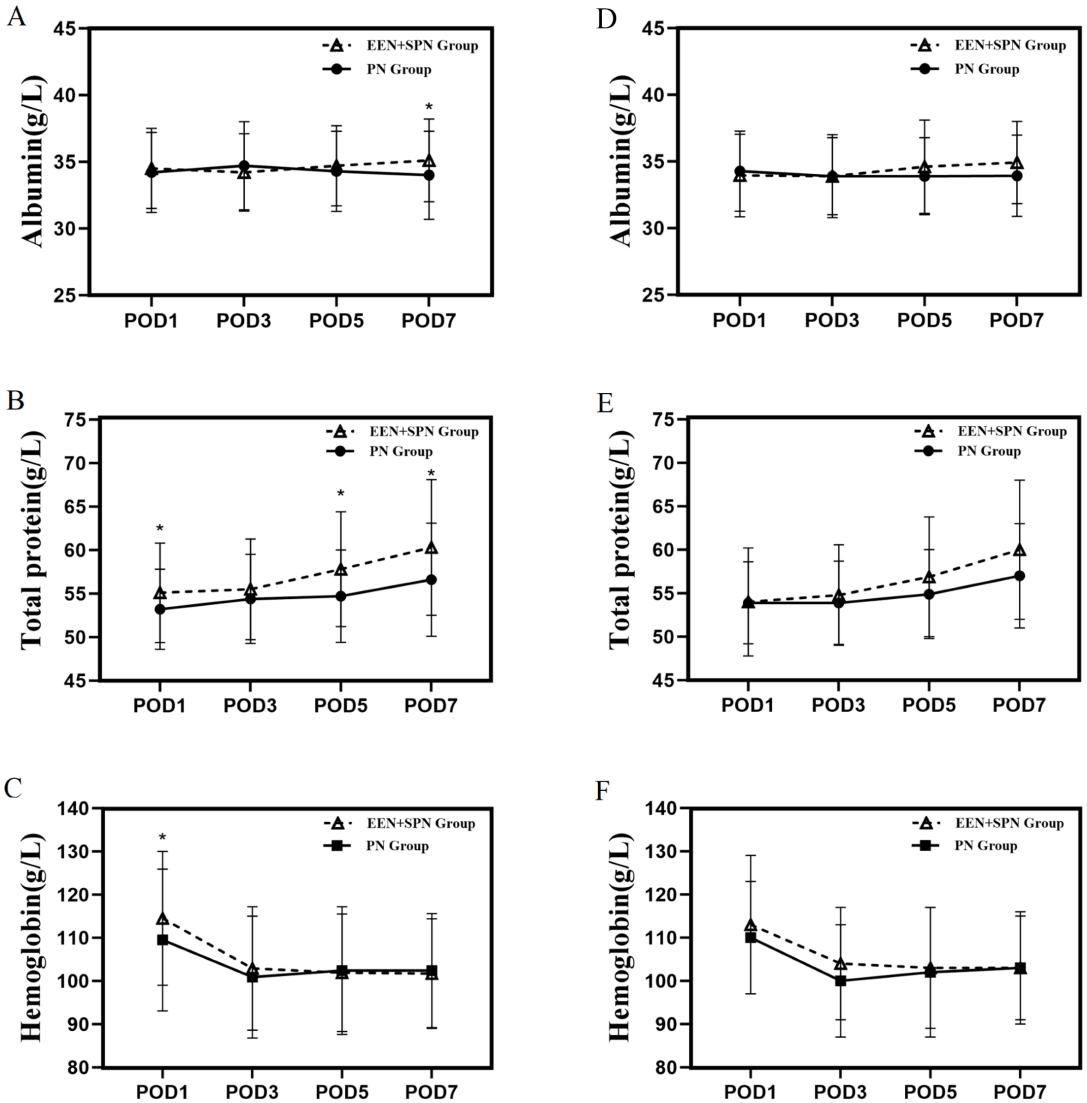
**(A)** Before PSM, mean albumin on days 1, 3, 5, 7 postoperatively; **(B)** before PSM, mean total protein on days 1, 3, 5, 7 postoperatively; **(C)** Before PSM, mean hemoglobin on days 1, 3, 5, 7 postoperatively. **(D)** After PSM, mean albumin on days 1, 3, 5, 7 postoperatively. **(E)** After PSM, mean total protein on days 1, 3, 5, 7 postoperatively. **(F)** After PSM, mean hemoglobin on days 1, 3, 5, 7 postoperatively. *There was significant difference between the EEN + SPN and PN group.

The trends in postoperative nutritional indicators for both groups are shown in Figure 4. Mean (SD) POD 1 TP (55.2 [5.7] *vs*. 53.4 [4.7], *p* = 0.010), Hb (114.3 [15.6] *vs.* 108.9[14.6], *p* = 0.005); mean (SD) POD 5 TP (57.7 [6.7] *vs.* 54.9 [5.4], *P* < 0.001); mean (SD) POD 7 TP (60.6 [8.0] *vs.* 56.6[6.6], *p =* 0.008), Alb (35.0 [3.0] *vs.* 33.9 [3.4], *P* < 0.001), were significantly higher in the EEN + SPN group than in the PN group. However, after PSM, none of the nutritional indicators showed statistically significant differences between the two groups. (All nutritional indicator analyses are shown in Supplementary Table S4.)

## Discussion

In this retrospective study, EEN + SPN was associated with reduced postoperative severe complications in patients undergoing PD. In contrast, the serum levels of Alb and TP did not differ significantly between groups after matching; the apparent elevations observed in unmatched analyses were not retained post-matching.

PD is a highly invasive surgery associated with a significant risk of both overall and severe postoperative complications (23). In our single-center retrospective cohort study, we observed that the rate of severe postoperative complications was 10.1%, which aligns with findings from previous research (24). Notably, there were no recorded cases of postoperative mortality, indicating that PD can be performed safely at our center. DRM is prevalent among patients undergoing PD, with a reported prevalence of 80%. Many patients with pancreatic tumors experience considerable weight loss due to the nature of the tumors and gastrointestinal-associated gastrointestinal symptoms, including decreased appetite. These patients face inadequate nutrient intake for up to 10 days postoperatively, while in a state of physiological stress (25), which can contribute to malnutrition and elevate the risk of postoperative complications. Hence, it is crucial to identify effective nutritional interventions to address the nutritional deficiencies of patients in the early postoperative phase following PD.

Nutritional support can be delivered through both enteral and parenteral routes following PD. Traditionally, feeding for patients undergoing PD was initiated only after the return of bowel movements or flatus. However, this practice lacks an evidence-based foundation. In recent years, the ERAS concept has gained increasing acceptance in the context of PD. Based on the ERAS protocols, early oral feeding has been proven to be safe, although patients often fail to meet their energy and protein requirements adequately (26). Over the past decade, numerous clinical studies have reported and recommended the benefits of perioperative EEN as a safe and effective approach. Researchers believe that EEN is considered more physiologically appropriate as it helps maintain intestinal nutritional and immune function without increasing the risk of delayed gastric emptying or postoperative pancreatic fistula. Additionally, EEN has been shown to reduce the length of hospital stay (27–29). Consequently, EEN remains commonly recommended in clinical practice following PD. Historically, PN was the most commonly used and preferred method of postoperative care, but it is associated with risks such as catheter-related infections and sepsis, which in severe instances may be fatal. Nevertheless, a meta-analysis has demonstrated that PN significantly decreased mortality rates in patients with preoperative malnutrition (30, 31). According to a survey conducted by the Chinese Expert Consensus on Perioperative Pancreatic Surgery (32), PN continues to be the most frequently selected postoperative nutritional approach by Chinese surgeons, with approximately 77.3% using PN and 22.7% using SPN (33). A high-quality randomized controlled trial and a retrospective cohort study have demonstrated that EEN combined with SPN achieved energy target requirements and improved clinical prognosis rapidly (34, 35). Based on these findings, EEN combined with SPN appears to be a better postoperative nutritional support method compared to PN.

Severe postoperative complications can lead to unfavorable prognoses, prolonged hospitalizations, increased financial burdens, readmissions, and even death. Alb, TP, and Hb are well-established indicators of nutritional status. A reduction in the levels of these biomarkers indicates varying degrees of malnutrition (36–38). Previous studies (34, 39, 40) have indicated that the combination of EEN and SPN can provide sufficient nutrients and improve nutritional status—an effect potentially attributable to the EEN + SPN strategy’s ability to enhance energy supply. In the current study, however, Alb and TP levels were significantly higher in the EEN + SPN group compared to the PN group before matching; these differences lost statistical significance after PSM. This suggests that the initial discrepancy may have been influenced by baseline confounding factors. The absence of significant between-group differences in Alb and TP after matching implies that the apparent superiority observed in the PSM analysis may have been driven by baseline and perioperative imbalances. Therefore, these laboratory parameters should be interpreted as process indicators susceptible to short-term perioperative influences, rather than definitive endpoints of nutritional benefit.

The present study has several limitations. First, it is a retrospective study with propensity score matching analysis, which may be affected by selection bias and information bias, and cannot completely exclude the potential impact of confounding variables. Second, the study was a single-center study with a small sample size, which may limit the generalizability of the findings to broader populations. Therefore, large-scale randomized controlled trials are warranted to validate these findings. Future research should focus on comparing different EN formulas and evaluating the timing of nutritional support initiation to identify the most effective strategies for optimizing postoperative recovery. These efforts aim to further refine perioperative nutritional management in patients undergoing PD.

## Conclusion

In summary, this retrospective study indicates that EEN + SPN is a safe and feasible nutritional strategy for at-risk patients following PD. Our findings indicate that this approach is associated with a significantly lower incidence of severe postoperative complications, underscoring its potential value in optimizing clinical outcomes for this patient population.

## Data Availability

The raw data supporting the conclusions of this article will be made available by the authors, without undue reservation.
