# Supplementary material for: Early enteral nutrition combined with supplemental parenteral nutrition vs. total parenteral nutrition after pancreaticoduodenectomy: a retrospective and propensity score-matched analysis of postoperative complications
Source: Front Nutr. 2025 Nov 27;12:1606500. doi: 10.3389/fnut.2025.1606500 (PMC12696965; doi:10.3389/fnut.2025.1606500)
Supplement: Supplementary file 1 [file Data_Sheet_1.docx]

## SUPPLEMENTARY TABLES

### Table S1 Patient Characteristics Before and After propensity score-weighting analysis

| **Variables** | **Before PS matching** | | ***P*** | **SMD** | **After PS matching** | |  |  | **After PS Weighting** | |  |  |
| --- | --- | --- | --- | --- | --- | --- | --- | --- | --- | --- | --- | --- |
|  | **EEN+SPN**  **(n=116)** | **PN**  **(n=132)** |  |  | **EEN+SPN**  **(n=59)** | **PN**  **(n=59)** | ***P*** | **SMD** | **EEN+SPN**  **(n=237)** | **PN**  **(n=253)** | ***P*** | **SMD** |
| Age (mean±SD), years | 65.5±9.5 | 67.3±9.9 | 0.136 | 0.190 | 66.6±9.0 | 66.1±10.4 | 0.769 | 0.054 | 66.3±9.7 | 66.5±9.4 | 0.894 | 0.022 |
| Gender, n (%)  Male  Female | 71  45 | 72  60 | 0.289 | 0.135 | 36  23 | 31  28 | 0.457 | 0.172 | 144  93 | 151  102 | 0.889 | 0.024 |
| BMI (mean±SD), kg/m^2^ | 22.8±3.0 | 22.4±2.8 | 0.365 | 0.116 | 22.3±2.9 | 22.2±2.8 | 0.794 | 0.048 | 22.6±3.2 | 22.5±3.0 | 0.885 | 0.029 |
| GLIM, n (%) | 65(56.0%) | 91(68.9%) | 0.036 | 0.269 | 38(64.4%) | 40(67.8%) | 0.846 | 0.072 | 154(65.0%) | 179(70.7%) | 0.474 | 0.122 |
| DM, n (%) | 24(20.7%) | 27(20.5%) | 0.964 | 0.006 | 15(25.4%) | 11(18.6%) | 0.505 | 0.164 | 50(21.1%) | 53(20.9%) | 0.989 | 0.002 |
| HBP, n (%) | 57(49.1%) | 67(50.8%) | 0.799 | 0.032 | 31(52.5%) | 32(54.2%) | 1.000 | 0.034 | 123(51.9%) | 129(50.9%) | 0.921 | 0.017 |
| Jaundice, n (%) | 52(44.8%) | 59(44.7%) | 0.984 | 0.003 | 28(47.5%) | 31(52.5%) | 0.713 | 0.102 | 111(46.8%) | 121(47.8%) | 0.928 | 0.016 |
| PBD, n (%) | 31(26.7%) | 38(28.8%) | 0.717 | 0.046 | 16(27.1%) | 21(35.6%) | 0.427 | 0.183 | 65(27.4%) | 72(28.4%) | 0.861 | 0.029 |
| TP(mean±SD), g/L | 64.3±6.4 | 64.3±5.6 | 0.939 | 0.010 | 64.2±7.2 | 64.0±5.9 | 0.854 | 0.034 | 63.6±6.7 | 64.3±5.2 | 0.519 | 0.112 |
| Alb(mean±SD), g/L | 38.3±3.6 | 37.7±3.5 | 0.133 | 0.176 | 37.8±3.9 | 37.4±3.5 | 0.639 | 0.087 | 37.8±3.4 | 37.8±3.4 | 0.996 | 0.001 |
| PA(mean ± SD), mg/L | 199.9±59.6 | 188.1±58.6 | 0.134 | 0.148 | 192.2±58.8 | 184.6±52.8 | 0.459 | 0.137 | 193.6±58.8 | 192.9±56.7 | 0.948 | 0.012 |
| Hb(mean±SD), g/L | 122.2±17.6 | 120.3±22.7 | 0.460 | 0.093 | 120.2±18.1 | 120.7±26.3 | 0.964 | 0.008 | 120.6±17.5 | 121.7±23.1 | 0.722 | 0.055 |
| TB(median, IQR),μmol/L | 21.5(10.7,137.8) | 24.2(10.2,110.1) | 0.574 | 0.159 | 84.2±102.5 | 84.4±98.9 | 0.991 | 0.002 | 78.9±95.2 | 80.3±92.8 | 0.928 | 0.015 |
| DB(median, IQR),μmol/L | 10.5(2.2,95.1) | 12.0(2.4,77.8) | 0.754 | 0.178 | 54.9±71.5 | 54.9±67.8 | 0.999 | <0.001 | 51.7±66.9 | 52.4±64.4 | 0.947 | 0.011 |
| Pancreatic texture n (%)  Soft  Firm | 52  64 | 88  44 | <0.001 | 0.451 | 26  33 | 31  28 | 0.461 | 0.170 | 124  113 | 133  120 | 0.952 | 0.011 |
| Pathology, n (%)  PDAC  Non-PDAC | 99  17 | 108  24 | 0.456 | 0.095 | 50  9 | 48  11 | 0.806 | 0.090 | 45  192 | 48  205 | 1.000 | <0.001 |
| Vessel resection, n (%)  Yes  NO | 4(3.4%) | 22(16.7%) | <0.001 | 0.451 | 3(5.1%) | 5(8.5%) | 0.714 | 0.135 | 22(9.3%) | 25(9.8%) | 0.948 | 0.014 |
| Surgical method, n (%)  PD  PPPD | 106  10 | 70  62 | <0.001 | 0.947 | 49  10 | 50  9 | 1.000 | 0.046 | 175  62 | 181  72 | 0.819 | 0.045 |
| Operating time  ((mean±SD)), min | 271.7±73.6 | 351.9±101.3 | <0.001 | 0.907 | 291.2±82.3 | 303.3±106.7 | 0.483 | 0.130 | 307.3±96.1 | 306.1±105.7 | 0.957 | 0.011 |
| Blood loss volume  (median, IQR), ml | 300(200, 500) | 400(200, 600) | 0.114 | 0.171 | 427.1±300.5 | 472.2±316.9 | 0.440 | 0.046 | 455.7±345.2 | 463.2±306.5 | 0.907 | 0.023 |

BMI=body mass index; DM=diabetes mellitus; HBP= high blood pressure; PBD= preoperative biliary drainage; TP= Total Protein; Alb= albumin; PA= Prealbumin; Hb= hemoglobin; TB=total bilirubin; DB=direct bilirubin; EEN=early enteral nutrition; SPN=supplemental parenteral nutrition; TPN=[total parenteral nutrition](https://pubmed.ncbi.nlm.nih.gov/417412/); PD=pancreaticoduodenectomy; PPPD=pylorus-preserving pancreaticoduodenectomy; PDAC=pancreatic duct adenocarcinoma.

## Table S2 Comparison of postoperative complications in the unmatched and weighting group according to perioperative nutritional support modality.

|  | Before PS matching | |  | After PS matching | | After PS Weighting | | | |
| --- | --- | --- | --- | --- | --- | --- | --- | --- | --- |
|  | EEN+SPN  (n=116) | PN  (n=132) | *P* | EEN+SPN  (n=59) | PN  (n=59) | *P* | EEN+SPN  (n=237) | PN  (n=253) | *P* |
| Postoperative Complications | 76(65.5%) | 97(73.5%) | 0.221 | 42(71.2%) | 37(62.7%) | 0.434 | 168(70.9%) | 167(66.0%) | 0.516 |
| CD-I | 9(7.8%) | 13(9.8%) | 0.724 | 6(10.2%) | 2(3.4%) | 0.600 | 25(10.5%) | 18(7.1%) | 0.572 |
| CD-II | 63(54.3%) | 63(47.7%) | 0.364 | 33(55.9%) | 23(39.0%) | 0.097 | 135(57.0%) | 119(47.0%) | 0.251 |
| CD- III-V | 4(3.4%) | 21(15.9%) | 0.002 | 3(5.1%) | 12(20.3%) | 0.027 | 8(3.4%) | 28(11.1%) | 0.034 |
| CR-POPF | 20(17.2%) | 30(22.7%) | 0.283 | 13(22.0%) | 12(20.3%) | 1.000 | 36(15.2%) | 44(17.4%) | 0.667 |
| DGE | 20(17.2%) | 19(14.4%) | 0.539 | 12(20.3%) | 10(16.9%) | 0.813 | 43(18.1%) | 39(15.4%) | 0.692 |
| BL | 7(6.0%) | 17(12.9%) | 0.069 | 4(6.8%) | 7(11.9%) | 0.527 | 12(5.1%) | 33(13.0%) | 0.062 |
| CL | 14(12.1%) | 21(15.9%) | 0.386 | 10(16.9%) | 6(10.2%) | 0.420 | 45(19.0%) | 27(10.7%) | 0.186 |
| AP | 1(0.9%) | 4(3.0%) | 0.448 | 1(1.7%) | 2(3.4%) | 1.000 | 1(0.4%) | 5(2.0%) | 0.124 |
| PPH | 4(3.4%) | 10(7.6%) | 0.160 | 2(3.4%) | 4(6.8%) | 0.675 | 8(3.4%) | 19(7.5%) | 0.243 |
| Abdominal-infection | 40(34.5%) | 63(47.7%) | 0.035 | 20(33.9%) | 25(42.4%) | 0.448 | 90(38.0%) | 109(43.1%) | 0.548 |
| Pneumonia | 2(1.7%) | 3(2.3%) | 1.000 | 2(3.4%) | 2(3.4%) | 1.000 | 5(2.1%) | 4(1.6%) | 0.921 |
| Surgical site | 1(0.9%) | 4(3.0%) | 0.448 | 0(0%) | 2(3.4%) | 0.476 | 1(0.4%) | 10(4.0%) | 0.074 |
| Urinary tract infection | 1(0.9%) | 0(0%) | 0.948 | 1(1.7%) | 0(0%) | 1.000 | 1(0.4%) | 0(0%) | 0.309 |
| Bacteraemia | 3(2.6%) | 8(6.1%) | 0.185 | 2(3.4%) | 7(11.9%) | 0.165 | 5(2.1%) | 11(4.3%) | 0.244 |

CR-POPF=Clinically relevant postoperative pancreatic fistula (Grade B/ C); DGE=delayed gastric emptying; BL=biliary leakage; CL= Chylous fistula;A

P=acute pancreatitis; PPH=post-pancreatectomy hemorrhage.

**Table S4 AUCs and 95% CI**

| Response | Predictor | AUC | 95% CI |
| --- | --- | --- | --- |
| Complications | POD3EN | 0.550 | 0.439 - 0.661 |

**Table S4 Model information of RCS**

| Characteristics | Coefficient | SE | Statistic | *P*-value |
| --- | --- | --- | --- | --- |
| Intercept | 3.813 | 4.234 | 0.901 | 0.370 |
| POD3EN | -0.485 | 0.608 | -0.798 | 0.427 |
| POD3EN' | 3.095 | 3.197 | 0.968 | 0.335 |
| POD3EN'' | -9.851 | 10.761 | -0.915 | 0.362 |

Table S4 Threshold efect analysis of POD3EN on Complications

|  | OR (95% CI) | *P*-value |
| --- | --- | --- |
| Fitting by standard Logistic regression model | 1.06 (0.84, 1.33) | 0.611 |
| Fitting by piecewise Logistic regression model (break-point = 8.22) |  |  |
| POD3EN < 8.22 | 0.94 (0.60, 1.47) | 0.784 |
| POD3EN ≥ 8.22 | 1.18 (0.79, 1.76) | 0.415 |
| Log likelihood ratio |  | 0.509 |

## Table S4. Mean total protein, albumin and hemoglobin on days 1, 3, 5, 7 postoperativly.

|  | **EEN+SPN(N=116)** | **PN(N=132)** | ***P*-Value** |
| --- | --- | --- | --- |
| **Nutrirional indicators** | | | |
| **POD1** | | | |
| Alb (g/L) | 34.4±3.1 | 34.2±3.1 | 0.540 |
| TP(g/L) | 55.2±5.7 | 53.4±4.7 | 0.010 |
| Hb(g/L) | 114.3±15.6 | 108.9±14.6 | 0.005 |
| **POD3** |  |  |  |
| Alb (g/L) | 34.1±3.0 | 34.5±3.4 | 0.306 |
| TP(g/L) | 55.4±6.0 | 54.5±5.2 | 0.216 |
| Hb(g/L) | 102.9±14.6 | 100.7±14.0 | 0.196 |
| **POD5** |  |  |  |
| Alb (g/L) | 34.5±3.0 | 34.3±3.2 | 0.577 |
| TP(g/L) | 57.7±6.7 | 54.9±5.4 | <0.001 |
| Hb(g/L) | 101.0(91.8,109.2) | 101.0(92.0,112.5) | 0.608 |
| **POD7** |  |  |  |
| Alb (g/L) | 35.0±3.1 | 33.9±3.4 | 0.008 |
| TP(g/L) | 60.6±8.0 | 56.6±6.6 | <0.001 |
| Hb(g/L) | 101.6±12.9 | 101.8±13.0 | 0.894 |

TP, total Protein; Alb, albumin; Hb, hemoglobin.

## Table S4. After PSM, mean total protein, albumin and hemoglobin on days 1, 3, 5, 7 postoperativly.

|  | EEN+SPN(N=59) | PN(N=59) | P-Value |
| --- | --- | --- | --- |
| **Nutrirional indicators** | | | |
| **POD1** | | | |
| Alb (g/L) | 33.9±3.1 | 34.3±3.2 | 0.591 |
| TP(g/L) | 54.0±6.2 | 53.9±4.7 | 0.907 |
| Hb(g/L) | 113.0±16 | 110.0±13.0 | 0.345 |
| **POD3** |  |  |  |
| Alb (g/L) | 33.9±2.9 | 33.9±3.1 | 0.992 |
| TP(g/L) | 54.8±5.8 | 53.9±4.8 | 0.363 |
| Hb(g/L) | 104.0±13.0 | 100.0±13.0 | 0.153 |
| **POD5** |  |  |  |
| Alb (g/L) | 34.6±3.5 | 33.9±2.9 | 0.216 |
| TP(g/L) | 56.9±6.9 | 54.9±6.9 | 0.078 |
| Hb(g/L) | 103±14 | 102±15 | 0.706 |
| **POD7** |  |  |  |
| Alb (g/L) | 34.9±3.1 | 33.9±3.0 | 0.090 |
| TP(g/L) | 60±8 | 57.0±6.0 | 0.085 |
| Hb(g/L) | 103.0±12.0 | 103.0±12.0 | 0.948 |

TP, total Protein; Alb, albumin; Hb, hemoglobin.
